# Supplementary material for: The genome sequence of the Pine Beauty, Panolis flammea (Denis & Schiffermüller), 1775 (Lepidoptera: Noctuidae)
Source: Wellcome Open Res. 2025 Aug 15;10:444. [Version 1] doi: 10.12688/wellcomeopenres.24674.1 (PMC12441672; doi:10.12688/wellcomeopenres.24674.1)
Supplement: Peer review history

# Reviewer report: Reviewer response for version 1

**DOI:** 10.21956/wellcomeopenres.27189.r130117

**Published:** 2025-09-16

**Authors:** Sivasankaran Kuppusamy

**Affiliations:** 1 Loyola College, Chennai, India

A comprehensive chromosome-level genome of *Panolis flammea* (Denis & Schiffermuller, 1775) was sequenced, assembled and annotated using appropriate methods and softwares. The assembly contains two haplotypes with lengths of 709.26 Mb and 713.60 Mb, respectively. The sex chromosome Z was assigned based on the synteny to the genome of *Agriopis aurantiaria*.  

**Comments on the manuscript**

 In the title of the manuscript authors have given the species described year outside the parentheses like (Denis & Schiffermuller),1775. The species' descriptors' name and year can be given within the parentheses i.e., (Denis & Schiffermuller, 1775).

 The assembly contains two haplotypes with more or less equal megabses. One haplotype scaffolded up to chromosome level another one is annotated up to scaffolding level only. Is there any specific reason?

 The table 1 caption “Specimen details, sequencing platforms, and data yields are summarized in Table 1” was given two times in the methods. One may be deleted

 Sequence annotation details like number of protein-coding genes Gene transcripts, non-coding genes have not been given in the text.

 Page no 5 first column first paragraph “Thescaffolded” words were fused. The words can be separated

 The research article was well prepared and the manuscript meets the necessary scientific standard and is suitable for indexing

Are sufficient details of methods and materials provided to allow replication by others?

Yes

Is the rationale for creating the dataset(s) clearly described?

Yes

Are the datasets clearly presented in a useable and accessible format?

Yes

Are the protocols appropriate and is the work technically sound?

Yes

Reviewer Expertise:

Mitochondrial genome analysis of Noctuoidea moths

I confirm that I have read this submission and believe that I have an appropriate level of expertise to confirm that it is of an acceptable scientific standard.

# Reviewer report: Reviewer response for version 1

**DOI:** 10.21956/wellcomeopenres.27189.r130123

**Published:** 2025-09-16

**Authors:** Arun Arumugaperumal

**Affiliations:** 1 Department of Biotechnology, Rajalakshmi Engineering College, Chennai, Tamil Nadu, India

The data note describes the genome sequence of the moth *Panolis flammea*, also known as the Pine Beauty. The photograph of the remains of specimen used for sequencing is shown in figure 1. But the remains does not provide a clear image of the moth. A photograph of an alive moth might provide more information about its identity. The authors have used advanced sequencing technology and the final genome assembly size is 709.26 Mb spread across 31 chromosomes. Since this is a male specimen they have identified a Z chromosome. The moth has ZW sex system. It would have been better if the authors had chosen a female specimen for sequencing. The mitochondrial genome was also assembled and is of size 15.46 kb. BUSCO analysis shows that the completeness percentage is 98.8%. The annotations were supposed to be released in Ensembl but as of now it has not been released. The accession numbers of the INSDC databases were working correctly. The article can be indexed.

Are sufficient details of methods and materials provided to allow replication by others?

Yes

Is the rationale for creating the dataset(s) clearly described?

Yes

Are the datasets clearly presented in a useable and accessible format?

Yes

Are the protocols appropriate and is the work technically sound?

Yes

Reviewer Expertise:

Genomics; Bioinformatics

I confirm that I have read this submission and believe that I have an appropriate level of expertise to confirm that it is of an acceptable scientific standard.
